# Bio-Based Silica-Reinforced Chitosan/Collagen Thermogels: Synthesis, Structure, and Rheological Behavior

**DOI:** 10.3390/polym17182476

**Published:** 2025-09-13

**Authors:** Amakorn Poommoon, Piyanut Nookong, Santamon Pengoubol, Panjaporn Wongwitthayakool

**Affiliations:** 1 Department of Prosthodontics, Faculty of Dentistry, Thammasat University, Pathumthani 12120, Thailand; poommoon@tu.ac.th; 2 Department of Oral Biology, Faculty of Dentistry, Thammasat University, Pathumthani 12120, Thailand; piyanut.noo@dome.tu.ac.th; 3 Department of Periodontology, Faculty of Dentistry, Thammasat University, Pathumthani 12120, Thailand; santamon@tu.ac.th

**Keywords:** chitosan, collagen, silica, rice husk ash, thermosensitive hydrogel, FTIR characterization, rheological properties, porosity and microstructure

## Abstract

Silica-reinforced chitosan/collagen hydrogels are useful for biomedical applications. In this study, thermosensitive chitosan/collagen hydrogels were prepared with different amounts of rice husk ash-derived silica (RHA-Si). Fourier-transform infrared (FTIR) spectroscopy was used to analyze the chemical structure. Results showed that adding RHA-Si did not change the main chemical groups but caused slight shifts, indicating physical interactions. Micro-Computed Tomography (Micro-CT) revealed that RHA-Si altered the shape and size of the pores in the hydrogel. The pore structure became more spherical at certain RHA-Si levels, but not consistently. Rheological tests showed that increasing RHA-Si made the hydrogel stiffer and reduced the gelation time. However, the hydrogel weakened under high strain due to broken physical bonds. Compression tests indicated that low RHA-Si (1% *w*/*v*) improved the hydrogel’s strength during small deformations. In contrast, the hydrogel was less resistant to compression at higher RHA-Si levels (2–3% *w*/*v*). In summary, adding RHA-Si can improve the structure and strength of chitosan/collagen hydrogels, but excessive RHA-Si may reduce flexibility. The RHA-Si content should be adjusted to match the intended application of the hydrogel.

## 1. Introduction

Injectable hydrogels have extensive applications in regenerative medicine due to their high tissue-like water content, ability to homogeneously encapsulate cells, efficient mass transfer, and easily manipulated physical properties [1]. When used at the defect site, injectable hydrogels deliver the loaded content and then form a gel through transitional properties when a response to a stimulus changes the physical and/or chemical properties. Different types of injectable scaffolds, such as microparticles, hydrogel, nano-composite films, and nanoparticles, have been developed using biomaterials such as natural and synthetic polymers [2].

Natural polymers used include chitosan, cellulose, gelatin, xyloglucan, alginate, hyaluronic acid, chondroitin sulfate, carrageenan, dextran, and pullulan and their respective derivatives [3,4,5]. Synthetic polymer materials comprise poly-N-isopropylacrylamide (PNIPAAM), poly(ethylene oxide)-b-poly(propylene oxide)-b-poly(ethylene oxide) (PEO/PPO/PEO) block copolymers, poly(ethylene oxide)-b-poly(D, L-lactic acid-co-glycolic acid)-poly(ethylene oxide) (PEO/PLGA/PEO) tri-block copolymers, and amphiphilic tri-block copolymers composed of PEO and poly-ε-caprolactone (PCL) (PEO/PCL/PEO) [6,7]. Natural-based hydrogels have been extensively researched among these two types due to their outstanding biological features [8]. Chitosan has many applications due to its unique physicochemical and biological properties, including biocompatibility, biodegradability, nontoxicity, inertness, strong affinity for proteins, and intrinsic antibacterial activity. Other valuable properties encompass moldability into various forms, compatibility with a wide range of delivery materials, drug-carrying capacity, a cationic nature, and the ability to engage in electrostatic interactions with anionic glycosaminoglycans (GAG) and proteoglycans, as well as other negatively charged species. However, significant drawbacks of hydrogels made from collagen and/or chitosan include their limited mechanical strength and rapid biodegradation [9,10,11,12,13].

One of the strategies used to improve the mechanical properties of the component hydrogel network is the addition of clays and nanoparticles (NPs) to polymer solutions that may act as cross-linkers [14]. This strategy may allow the formation of composite gels that are mechanically stronger due to the integration of entities supporting dissipative mechanisms [15]. These nanoparticles crosslink the hydrogel or adsorb polymer chains by being entrapped within the hydrogel network. 

Silica-based nanoparticles have been used extensively for various biomedical applications due to their excellent elastic modulus, strength, and toughness properties. They provide a prime micro-structural design model for developing new materials. Silica possesses a large surface area and smooth nonporous surface, encouraging intense physical contact between the filler and the polymer matrix [16]. Recently, major efforts have been made toward using silica nanoparticles to fabricate mechanically strong nanocomposite hydrogel networks [17,18,19]. The hypothesis proposed by researchers is that strong polymer/nanofiller interactions might facilitate the formation of non-covalent or pseudo-crosslinks, thereby contributing to improvements in polymer properties [20].

However, conventional silica fillers are predominantly sourced from non-renewable minerals and involve energy-intensive extraction processes, raising concerns over sustainability and environmental impact [21]. Rice husk ash-derived silica (RHA-Si), obtained from the abundant agricultural waste of rice husks, provides a renewable and eco-friendly alternative with significant economic and environmental advantages [22,23]. It has been found that the silica content in rice husk ash, which is prepared by preliminary leaching of rice husks with a solution of hydrochloric acid before their combustion, can be as high as 99.5% [24]. Additionally, the physicochemical properties of RHA-Si, such as its specific surface area, porosity, and surface chemistry, contribute to enhanced interactions within the composite matrix, leading to mechanical strength comparable to conventional silica fillers [25,26]. This study uses the advantages of RHA-Si to support the development of sustainable composites. It offers a new perspective on choosing fillers that combine strong performance with environmental responsibility.

Studies on RHA-Si have shown varying effects on different polymers, with its reinforcing ability depending on the type of matrix and the quality of polymer–filler interactions. Fuad et al. [27] studied the impact of silica obtained from rice husk ash (RHA-Si) on the mechanical properties of polypropylene. The tensile strength, elongation at break, and impact strength were found to be decreased. The results were explained by poor polymer–filler interaction between polypropylene and RHA-Si. In the study by Siriwardena et al. [28], the effect of RHA-Si on the physical properties of ethylene-propylene-diene rubber (EPDM) were investigated. It was concluded that RHA-Si could not reinforce EPDM, although it did not decrease the physical properties of the EPDM. On the other hand, the study by Saowapark et al. found that the mechanical properties of deproteinized natural rubber could be improved by adding RHA-Si [29]. However, the RHA-Si added to dental materials, such as acrylic resin denture bases, could increase flexural strength, and polymer–filler interaction was used as the explanation [30]. Overall, these findings suggest that the performance of RHA-Si as a filler is highly material-dependent, highlighting the importance of matrix compatibility in achieving reinforcement.

Most of these studies have focused on synthetic or structural polymers, while little attention has been given to biopolymer-based systems for biomedical applications. This work incorporates RHA-Si into a chitosan/collagen thermogel, an injectable and biodegradable material, to improve its mechanical stability and potential bioactivity. This approach represents a new direction, repurposing RHA-Si from an industrial filler to a sustainable and biofunctional component for advanced biomedical materials.

A thermosensitive hydrogel is a temperature-responsive material that undergoes a sol–gel transition when exposed to physiological temperature, allowing injection in liquid form and subsequent gel formation in situ [31,32,33]. The term thermogel is often used to emphasize this gelation behavior at body temperature, which is particularly useful for biomedical applications such as drug delivery and tissue engineering [34,35]. In this system, β-glycerophosphate (β-GP) was employed as a pseudo-crosslinking agent to induce the thermosensitive gelation of chitosan. β-GP works by neutralizing the positive charges of chitosan and enhancing hydrogen bonding with collagen, enabling the hydrogel to remain injectable at room temperature and to form a stable gel at body temperature [36]. The overall objective of this research was to support the pseudo-crosslinking hypothesis using chitosan/collagen thermogels with RHA-Si as the model system and to investigate the effect of RHA-Si content on the chemical structure, morphology, porosity, and rheological and mechanical behavior of the thermogel.

## 2. Materials and Methods

### 2.1. Preparation of Rice Husk Ash Silica (RHA-Si)

Rice husk, obtained from a commercial rice mill in Ban Pong District, Ratchaburi Province, Thailand, in March 2023, was washed with distilled water and then air-dried at room temperature. The washed rice husk was refluxed with 1 M hydrochloric acid (HCl; RCI Labscan, Bangkok, Thailand) at 90 °C for an hour. After that, the HCl solution was wholly removed from the rice husk with distilled water. The refluxed rice husk was dried overnight in an oven at 60 °C and then burnt in an electric furnace at 700 °C for 5 h. RHA-Si was finally obtained in the form of white ash and was ground into a powder. The elemental composition and crystal structure of RHA-Si were then investigated with a wavelength-dispersive X-ray fluorescence spectrometer (WDXRF; ARL Perform’X; Thermo Fisher Scientific, Ecublens, Switzerland) and X-ray diffractometer (XRD; D8 Advance; Bruker Corporation, Karlsruhe, Germany), respectively.

### 2.2. Preparation of Thermosensitive Chitosan/Collagen Hydrogels

Chitosan solution (2% *w*/*v*) was prepared by dissolving chitosan (medium molecular weight; Sigma-Aldrich, St. Louis, MO, USA) in 0.1 M acetic acid at room temperature under moderate mechanical stirring at a speed of 250 rpm for 24 h. An aqueous solution of atelocollagen (1%) was added to the prepared chitosan solution with a 75/25% volume ratio of chitosan/collagen. To prepare the nanocomposite thermogel, RHA-Si was added to the chitosan/collagen mixtures with various loadings of 0.5, 1, 2, and 3% *w*/*v*, and then an aqueous solution of β-glycerophosphate (β-GP; Sigma-Aldrich, St. Louis, MO, USA) was added dropwise to the mixture with 20% *w*/*v* in an ice bath. The prepared thermogel solution was adjusted to pH 7.2 ± 0.1 at 4 °C with 1 M NaOH/HCl and then stored at 4 °C to maintain a liquid state. The gelation of the thermogel was achieved by incubating the solution at 37 °C.

### 2.3. Chemical Characterization of Thermogels by Fourier-Transform Infrared (FTIR) Spectroscopy

The freeze-dried thermogels were prepared through lyophilization, a process in which ice is removed via sublimation under vacuum conditions for 48 h, to investigate the chemical functional group using an FTIR spectroscope (Nicolet is5; Thermo Scientific, Waltham, MA, USA) equipped with an ATR accessory. The spectra were recorded at room temperature in the wavenumber range of 600–4000 cm^−1^ with a resolution of 4 cm^−1^ by the assemblage of 32 scans.

### 2.4. Morphological and Porosity Analysis of Thermogels Using Micro-CT

The morphological structure of the freeze-dried thermogels was analyzed using Micro-Computed Tomography (SkyScan 1173; Bruker microCT, Kontich, Belgium) with a voxel size of 9 µm. The samples were scanned under optimized parameters to capture their morphology, followed by image reconstruction and processing to assess the porosities within the thermogel network. Additionally, the porosity of the prepared thermogels was quantified using the SKY SCAN.

### 2.5. Rheological Measurements

Rheological measurement was performed using a parallel-plate rheometer (HAAKE MARS; Thermo Scientific, Karlsruhe, Germany). A time sweep test was conducted to monitor the effect of RHA-Si concentration on the setting time of the thermogel, which was performed at a temperature of 37 °C, frequency of 1 Hz, and strain of 5%. The resultant storage modulus or elastic modulus (G′) and loss modulus or viscous modulus (G″) were recorded for 30 min. The strain sweep test (varying from 0.01 to 10%) was carried out to investigate the rheological behavior of the thermogels in a linear viscoelastic region (LVR) at 37 °C and a frequency of 1 Hz.

### 2.6. Mechanical Test

Compression force at a given displacement was used to evaluate the mechanical properties of the freeze-dried thermogel samples. The sample specimens were prepared in a plastic mold (5 mm diameter, 8 mm height). The thermogel samples’ compressive force was measured using a universal testing machine (AGS-X; Shimadzu Corporation, Kyoto, Japan) with a crosshead speed of 1 mm/min at displacements of 25, 50, and 75%, respectively.

### 2.7. Statistical Analysis

SPSS version 31 was used as the statistical software for data analysis. Statistical significance was assessed using one-way ANOVA and Tukey’s HSD test. *p*-values less than 0.05 were considered statistically significant. All experiments were performed in triplicate, and the measured values were averaged (*n* = 3).

## 3. Results and Discussions

### 3.1. Chemical Composition and Structure of Rice Husk Ash Silica RHA-Si

**Figure 1:**
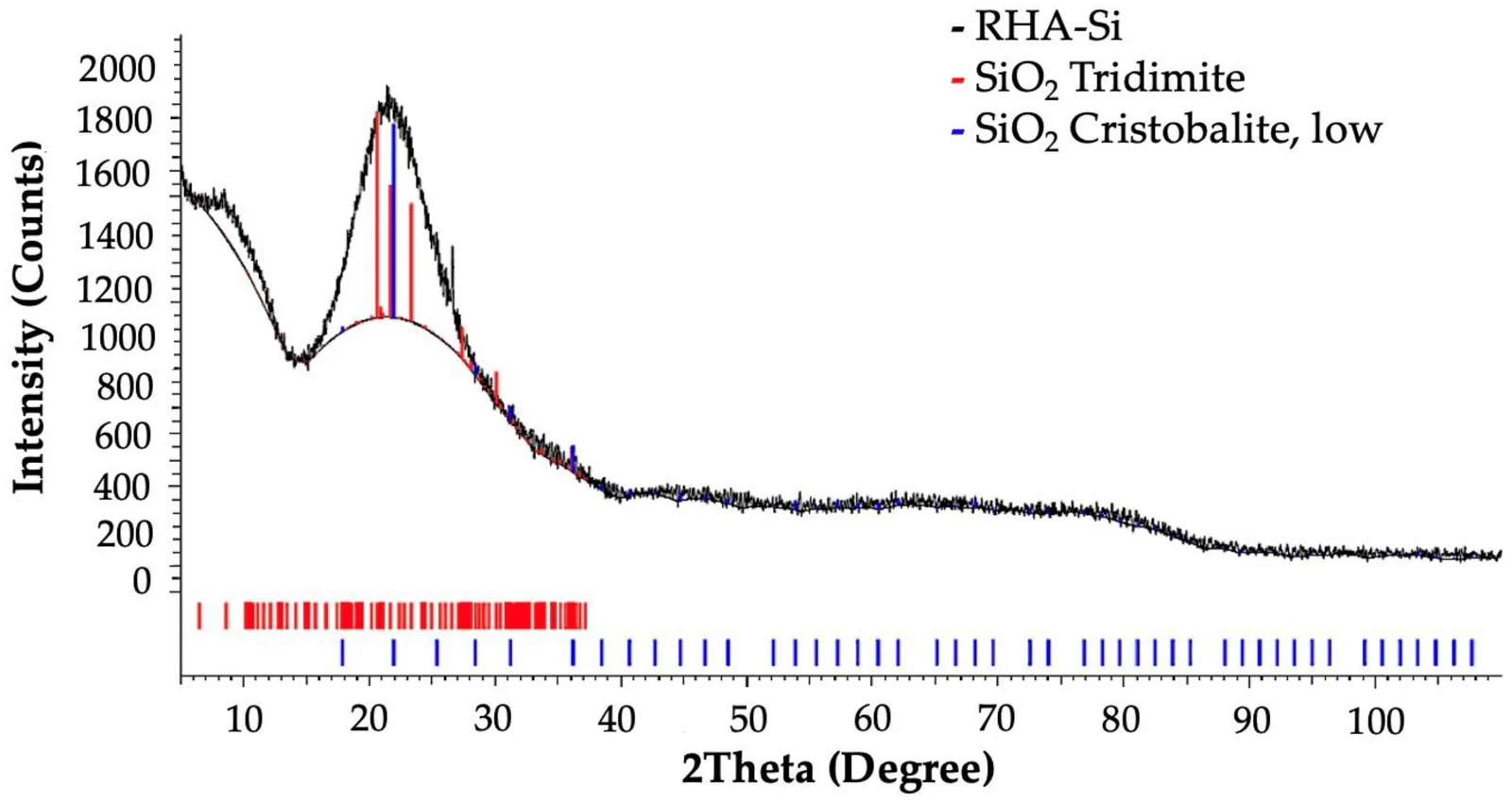
The XRD pattern of RHA-Si shows its amorphous structure.

The characterization results for RHA-Si are summarized in Table 1 and illustrated in Figure 1. Table 1 shows the chemical composition and relative percentages of elements in RHA-Si, as determined by X-ray fluorescence (XRF) analysis. Silicon dioxide (SiO_2_), or silica, was identified as the primary component, while various metallic impurities were also detected in smaller amounts.

The crystalline structure of RHA-Si was examined using X-ray diffraction (XRD), as shown in Figure 1. The XRD pattern exhibits a broad peak centered at 2θ = 22°, characteristic of amorphous silica. The absence of sharp, well-defined peaks indicates that RHA-Si lacks any significant crystalline structure [30].

### 3.2. Preparation and Chemical Characterization of Thermosensitive Chitosan/Collagen Hydrogels

**Figure 2:**
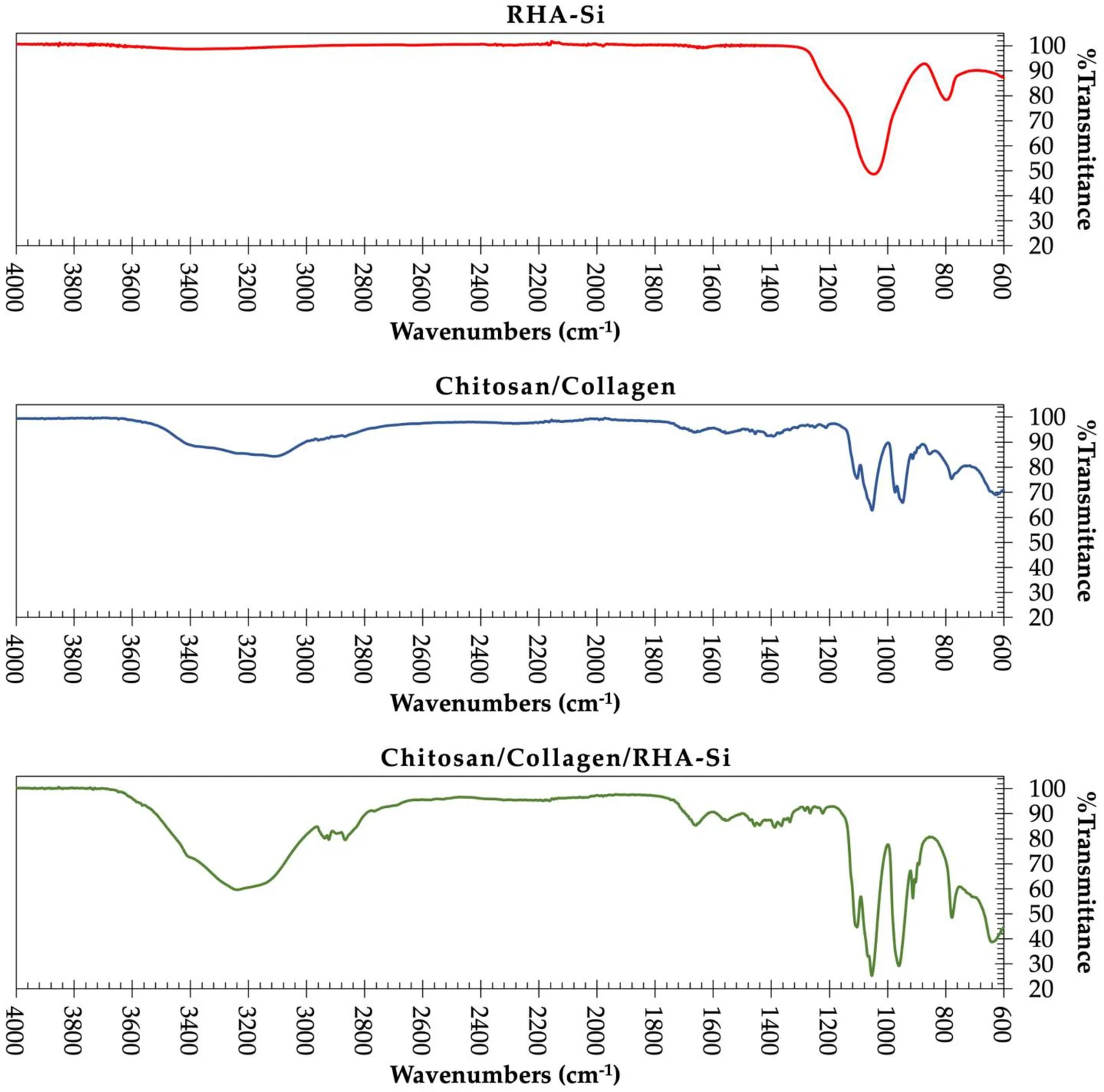
FTIR spectra of RHA-Si, chitosan/collagen thermogel, and chitosan/collagen/RHA-Si thermogel.

Thermosensitive chitosan/collagen hydrogels were prepared and characterized. The chemical groups of the thermogels were examined by FTIR, as presented in Figure 2. It is found that the FTIR spectra of RHA-Si commonly exhibit characteristic bands due to different vibrational modes of the Si-O and Si-OH bonds [37].

The 3397 cm^−1^ band represents the stretching vibration of hydroxyl (-OH) groups, which are present due to surface silanol groups (Si-OH) and adsorbed water on silica. The strong and broad band at 1045 cm^−1^ reveals a characteristic of the asymmetric stretching of the Si-O-Si network. The 796 cm^−1^ band corresponds to the symmetric stretching or bending vibrations of Si-O-Si bonds in silica.

The FTIR spectra of chitosan/collagen thermogels reveal distinct vibrational modes reflecting molecular interactions between the two biopolymers. The shoulder band at 3362 cm^−1^ corresponds to O-H stretching from hydroxyl groups in chitosan and collagen, enhanced by hydrogen bonding between the polymers. The 3230 and 3101 cm^−1^ sub-bands represent N-H stretching from amide groups in chitosan and collagen. The 2946 cm^−1^ band and 2866 cm^−1^ bands correspond to the asymmetric and symmetric stretching vibrations of CH_2_ and CH_3_ groups in the chitosan and collagen backbone. Amide-related bands include 1659 cm^−1^ (amide I, C=O stretching), 1564 cm^−1^ (amide II, N-H bending + C-N stretching), and 1301 cm^−1^ (amide III, C-N stretching + N-H deformation). Saccharide-specific bands appear at 1084 and 1052 cm^−1^, attributed to C-O-C and C-O stretching in chitosan, while the FTIR bands at 961 cm^−1^ and 945 cm^−1^ relate to pyranose ring vibrations in chitosan’s saccharide structure. The minor structural features, such as 779 and 622 cm^−1^, represent out-of-plane bending and deformation modes.

It is noticed that the addition of silica into chitosan/collagen thermogels does not significantly alter the primary functional groups, as indicated by the similar transmittance at the amide key bands. This suggests that adding silica into the thermogel does not represent a chemical interaction [38,39]. However, it could be observed that the 945 cm^−1^ band becomes a major band, replacing the 961 cm^−1^ band by adding silica into the thermogel. This is because the -NH and −OH of chitosan can interact with silica via hydrogen bonds [40], suppressing pyranose ring vibrations and reducing the 961 cm^−1^ band. Silica enhances collagen–silica hydrogen bonding, amplifying collagen-specific vibrations and causing the 945 cm^−1^ band to become apparent. This is consistent with collagen–silica electrostatic stabilization mechanisms observed in hybrid thermogels [41]. The effect of silica on thermogel could also be seen from the shift of the 622 cm^−1^ band to 636 cm^−1^, indicating structural modifications, likely due to interactions between silica and the biopolymer matrix. This band is associated with out-of-plane C-OH bending (chitosan) and C-C-O deformation (collagen), and its shift suggests that silica may be forming hydrogen bonds or electrostatic interactions with hydroxyl and amide groups, altering the local molecular environment [42]. Additionally, silica incorporation may influence the thermogel network’s rigidity, affecting the polymer chain’s vibrational modes. This shift confirms the successful integration of silica into the thermogel while maintaining the core molecular framework of the chitosan–collagen system.

### 3.3. Morphological and Porosity of Thermosensitive Chitosan/Collagen Hydrogels 

**Figure 3:**
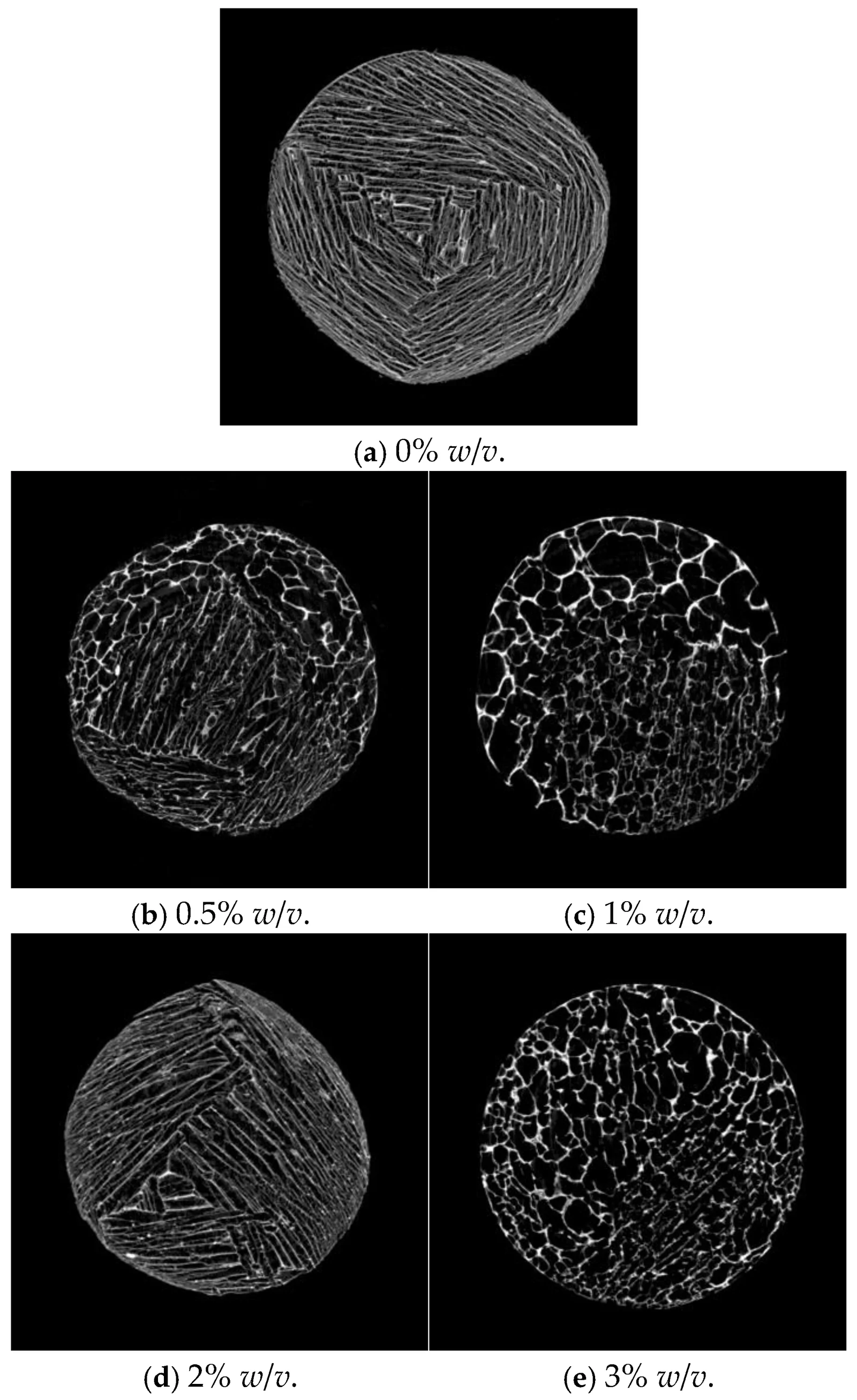
Morphology of freeze-dried chitosan/collagen thermogels with various RHA-Si concentrations, captured from image reconstruction of micro-CT analysis (voxel size = 9 µm): (**a**) 0% *w*/*v*; (**b**) 0.5% *w*/*v*; (**c**) 1% *w*/*v*; (**d**) 2% *w*/*v*; (**e**) 3% *w*/*v*.

Figure 3 shows the cross-section images of the freeze-dried thermogels captured from image reconstruction of micro-CT analysis. This Figure illustrates that incorporating RHA-Si into the chitosan/collagen thermogel influences its porous structure. At 0% *w*/*v* RHA-Si, the freeze-dried thermogel exhibits predominantly rod-shaped pores, suggesting anisotropic pore formation likely due to the intrinsic alignment of the polymer matrix during fabrication [43]. During freeze-drying, anisotropic polymer alignment leads to elongated, tubular pores. Interestingly, adding RHA-Si at concentrations of 0.5%, 1.0%, and 3.0% *w*/*v* resulted in a noticeable transformation of pore shapes from tubular to more spherical pores. This suggests that RHA-Si may act as a structural modifier, promoting spherical pore geometry and isotropic pore formation, possibly by (i) increasing crosslink density via the interaction between silica’s hydroxyl groups with chitosan/collagen and (ii) limiting polymer mobility by increasing solution viscosity by adding solids [42,44].

However, at 2.0% *w*/*v* RHA-Si, the pore shape returned to a tubular form, indicating a non-linear effect of RHA-Si concentration on pore structure. The reversion to a tubular pore structure at 2% *w*/*v* RHA-Si in chitosan/collagen thermogels during freeze-drying can be speculated to arise from a threshold behavior related to silica agglomeration affecting the ice-templating process. At lower silica concentrations, RHA-Si particles likely remain well dispersed in the thermogel matrix, uniformly influencing ice crystal nucleation and growth, which predictably impacts pore morphology. However, at or above the 2% threshold concentration, silica particles may begin to agglomerate or form clusters. These agglomerates can disrupt the uniformity of ice crystal growth during freezing, leading to a non-linear change in pore structure.

Specifically, agglomeration can locally alter freezing kinetics or create heterogeneous nucleation sites [45,46]. This effect disrupts the normal directional growth of ice crystals responsible for templating pore morphology, leading to tubular rather than spherical or irregular pores. The ice-templating process is sensitive to the balance between nucleation and ice crystal growth rates, which can be affected by filler clusters that change local freezing conditions and solvent displacement pathways in the thermogel matrix. 

Additional factors might include changes in the viscosity or mechanical properties of the thermogel with this specific RHA-Si loading, which could influence how the polymer chains and RHA-Si particles rearrange before and during freezing. Such non-linear threshold phenomena are common in nanocomposite systems where filler–filler interactions shift from well-dispersed states to agglomerated networks, leading to sudden changes in physical properties and microstructure. 

**Figure 4:**
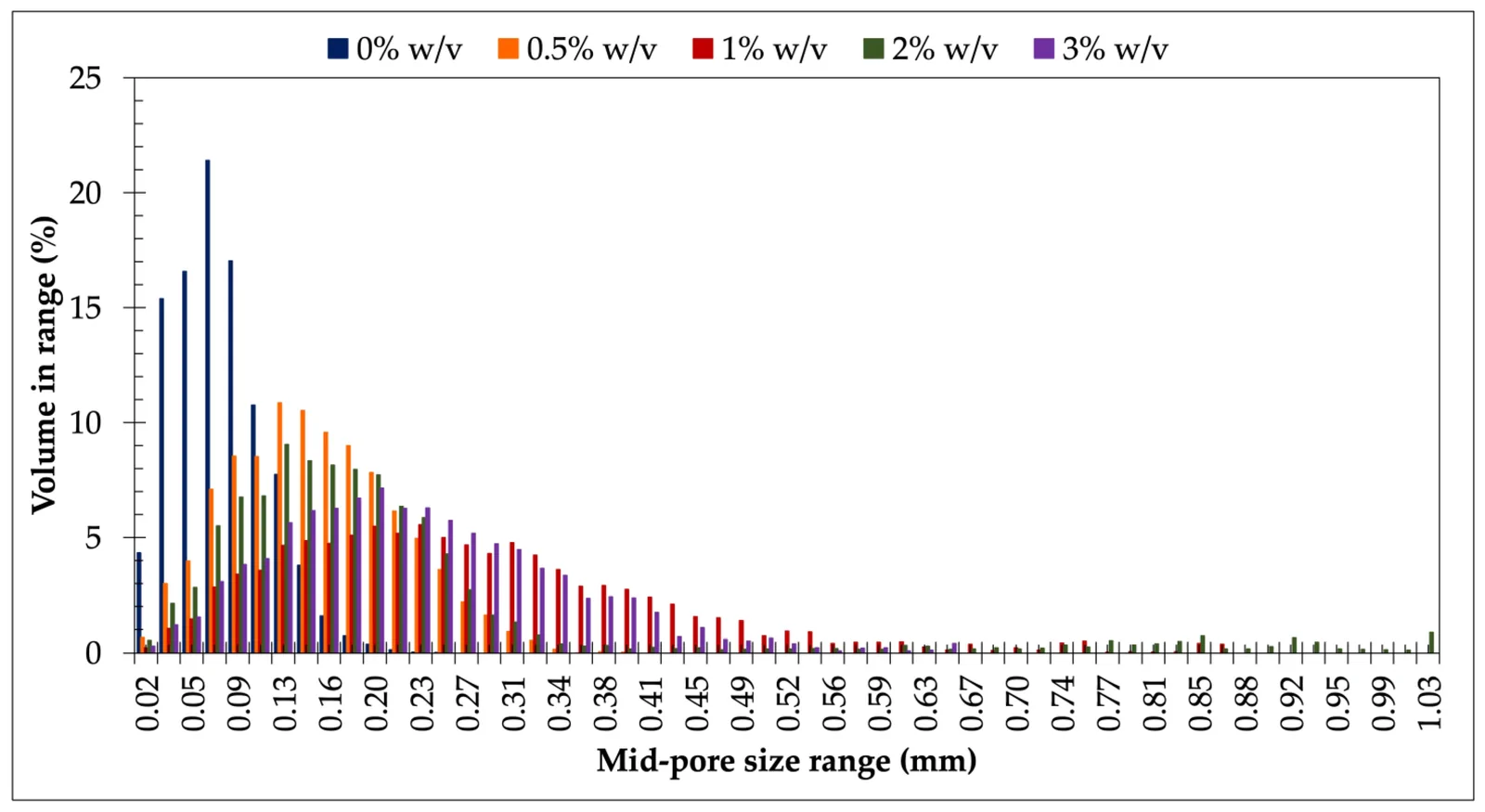
Pore size distribution of the freeze-dried chitosan/collagen thermogels with various RHA-Si concentrations.

Figure 4 illustrates the pore size and distribution of the freeze-dried thermogel filled with different RHA-Si concentrations. It is evident that at 0% *w*/*v* RHA-Si, the freeze-dried thermogel possesses the smallest pore size and the most uniformly distributed pores, as shown with the maximum mid-pore size range and standard deviation of structure separation (SD_St.Sp_) in Table 2, respectively: the SD_St.Sp_ represents the variability in pore-to-pore distances within the thermogel network; a higher SD_St.Sp_ indicates a more heterogeneous pore distribution, whereas a lower SD_St.Sp_ reflects a more uniform and homogeneous pore structure.

The effect of RHA-Si concentrations on the porosity and connectivity of the freeze-dried chitosan/collagen thermogels is presented in Table 3. The porosity increases and the connectivity density decreases as the RHA-Si concentration increases from 0 to 1.0, indicating a shift from small, well-connected tubular pores to larger, less interconnected spherical pores. Interestingly, at 2.0% RHA-Si, a partial recovery in connectivity density is observed, potentially due to the reformation of tubular pores. At 3.0% RHA-Si, porosity remains high, but connectivity is higher than in the 1% sample. This may reflect well-dispersed but relatively separate pores, possibly spherical. These results highlight the non-linear and concentration-dependent role of RHA-Si in modulating freeze-dried thermogel, which could impact mechanical integrity.

### 3.4. Rheological Behavior of Thermosensitive Chitosan/Collagen Hydrogels

The rheological behavior of chitosan/collagen thermogels with various concentrations of RHA-Si was investigated. The gelation time of the composite thermogels was assessed by rheological assays using the time sweep test at 37 °C. The gel point, which significantly increases the storage modulus (G′) and is markedly higher than the loss modulus (G″), marks the transition from a liquid state to a solid, gel-like state and is a good indicator of gelation formation. It is utilized to express the gelation time of the thermogel.

**Figure 5:**
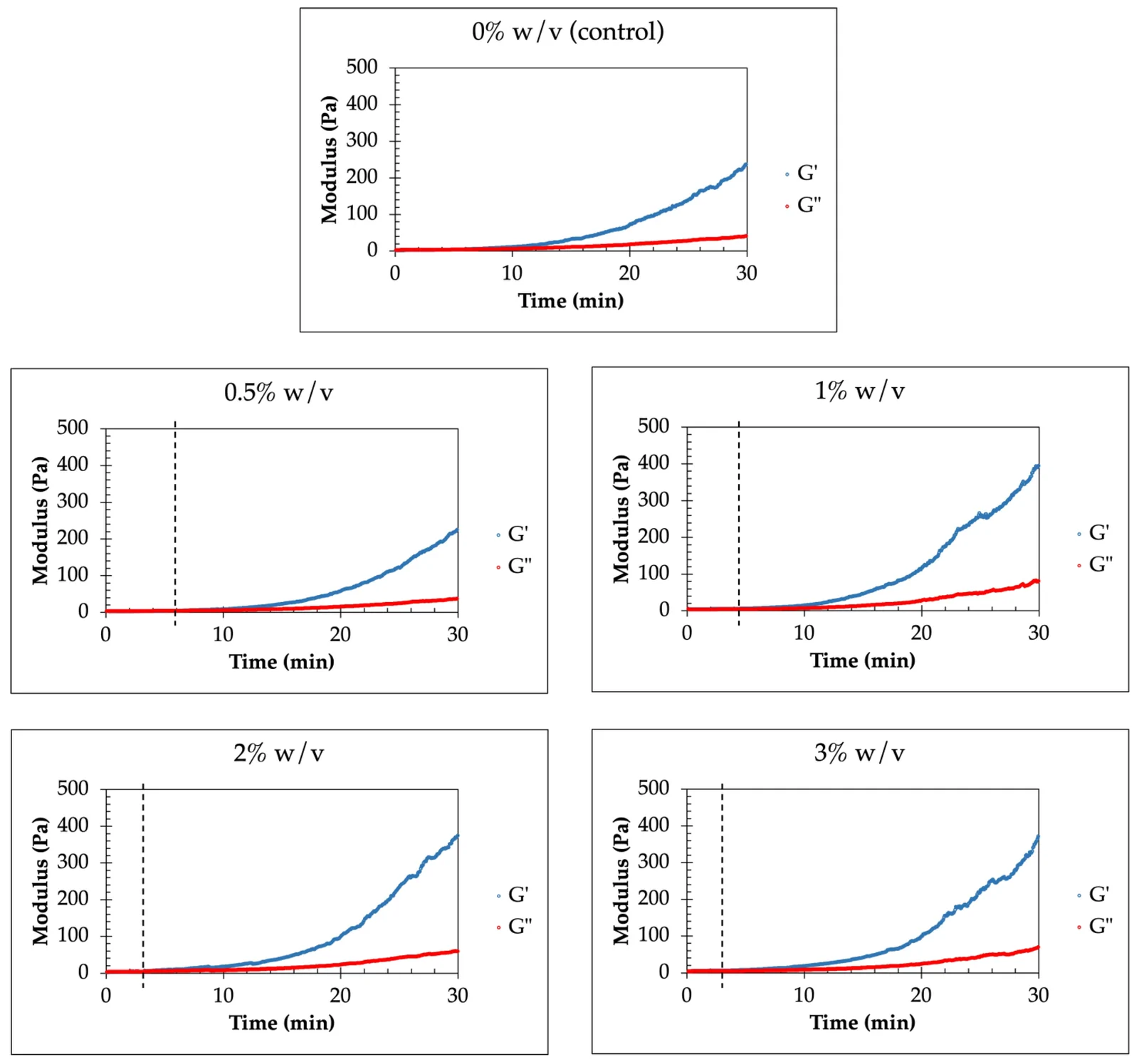
Storage moduls (G′) and loss modulus (G″) as a function of the time in chitosan/collagen thermogels with various RHA-Si concentrations.

From Figure 5, it can be seen that the thermogel’s gelation time (time at the dashed line) decreases with increasing RHA-Si loading. This is due to RHA-Si’s crosslink promotion. As discussed previously, RHA-Si contains silanol, which facilitates interactions with amine groups in chitosan or carboxyl/hydroxyl groups in collagen, promoting faster network stabilization.

**Figure 6:**
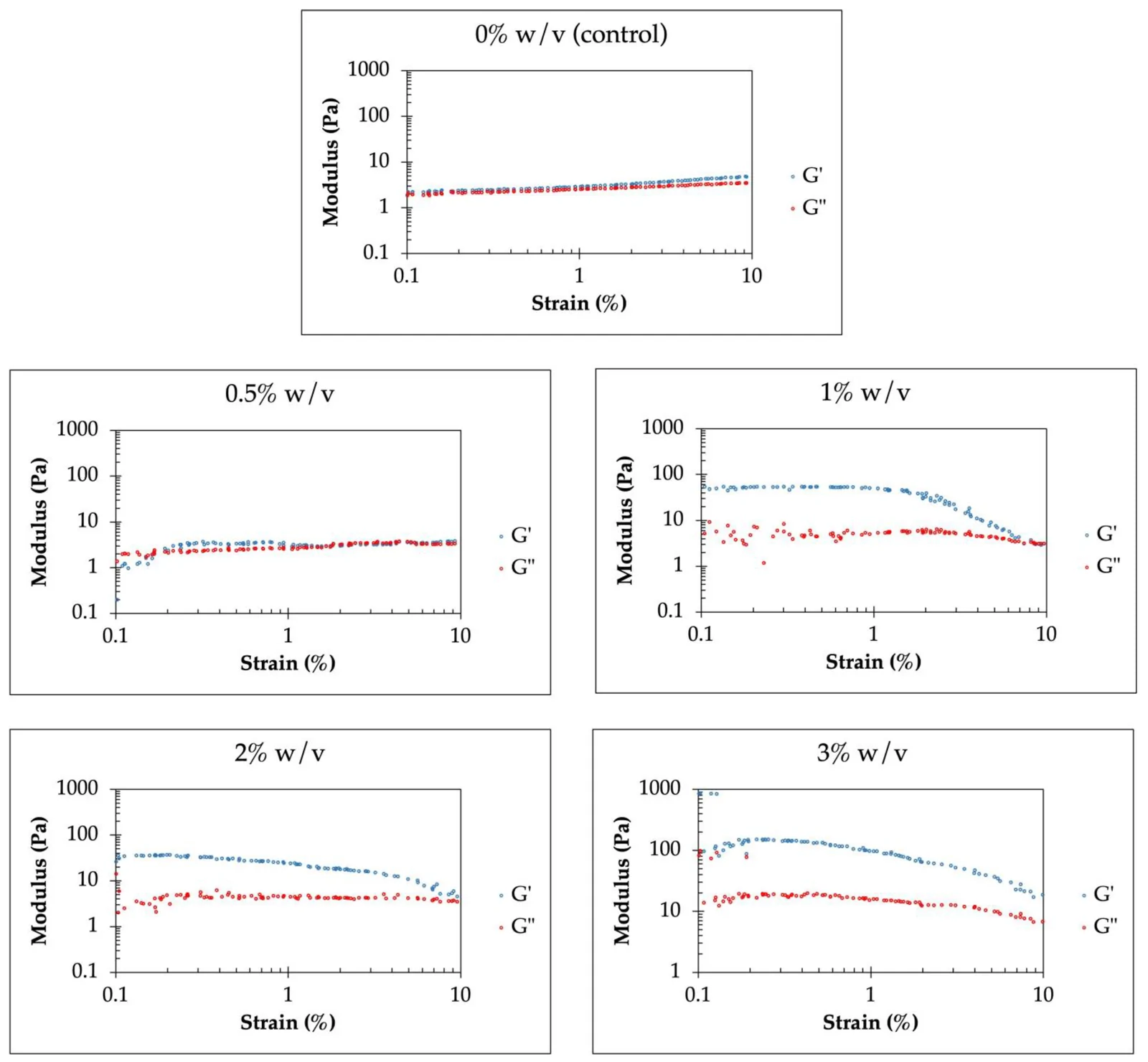
Storage modulus (G′) and loss modulus (G″) as a function of the strain in chitosan/collagen thermogels with various RHA-Si concentrations.

Figure 6 presents the G′ and G″ of the thermogel as a function of strain amplitude. Both the G′ and the G″ of a chitosan/collagen thermogel without RHA-Si remain nearly constant over a range of strain amplitudes, creating a well-defined linear viscoelastic region (LVR), indicating a good gel network. It is also found that the G′ is higher than the G″ across the examined strain amplitude range. This behavior can be attributed to the formation of a three-dimensional network due to strong physical interactions between chitosan and collagen associated with a physical crosslinker such as β-glycerophosphate (β-GP). The thermogel’s ability to retain a higher G′ than G″ throughout strain sweeps demonstrates that the elastic network dominates, with viscous dissipation playing a secondary role.

However, the increasing RHA-Si concentration leads to a higher G′ in the thermogel while simultaneously reducing the extent of the LVR. The increase in the storage modulus (G′) of chitosan/collagen thermogels with higher RHA-Si content can be attributed to the reinforcement effect imparted by the silica particles. Silica, as a rigid inorganic filler, enhances the mechanical strength of soft polymeric thermogels by undergoing physical interactions with the surrounding polymer chains. In particular, the silanol groups present on the surface of silica nanoparticles play a critical role in this reinforcement. As mentioned, these silanol groups can form hydrogen bonds with the hydroxyl, amine, or carboxyl groups of chitosan and collagen, leading to improved interfacial adhesion between the filler and matrix. This enhances stress transfer from the polymer to the filler and restricts the mobility of the polymer chains, thereby increasing the elasticity and G′ of the thermogel [47]. As the silica content increases, the network becomes more densely crosslinked.

The results show that the LVR is narrower with higher RHA-Si loading. This behavior is closely related to the Payne effect, which is commonly observed in filled polymers. The Payne effect describes a reduction in storage modulus with increasing strain amplitude, i.e., the structural breakdown in the filler–filler and filler–polymer network [48]. At higher strains, the physical interactions, especially hydrogen bonding between silanol groups of RHA-Si and polymer chains in chitosan/collagen thermogels, are disrupted, leading to a decrease in G′. However, it is noticeable that the G″ shows much less sensitivity to strain amplitude, which can be explained by the RHA-Si interaction via physical (non-covalent) bonds. At given strains, these bonds might partially break (reflected in the G′ drop). However, the polymer chains are still crosslinked or associated, so there is no significant increase or decrease in energy dissipation. Understanding this interplay is essential in designing thermogels with optimal mechanical and functional properties for biomedical applications.

### 3.5. Mechanical Properties

**Figure 7:**
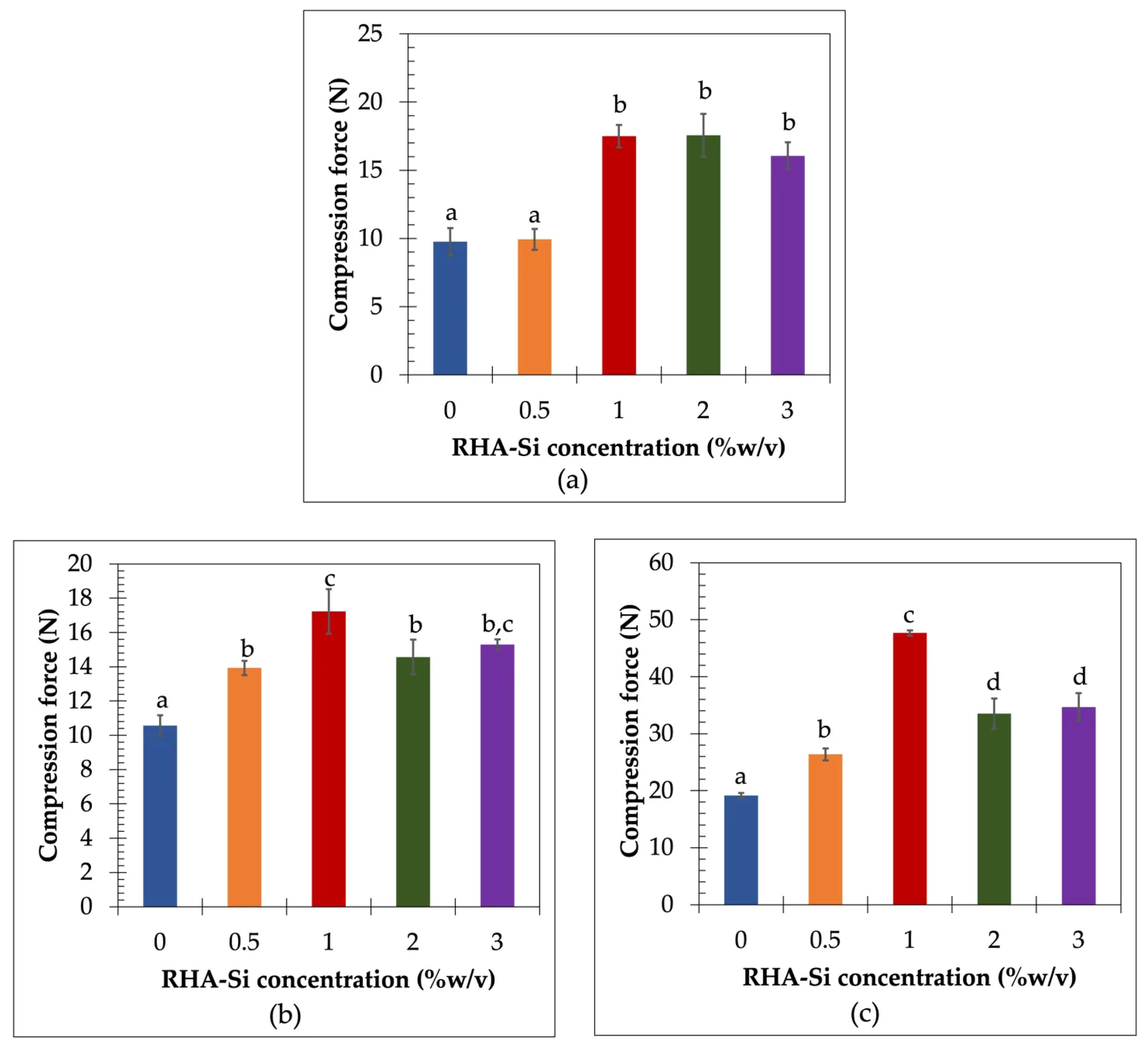
Compression of freeze-dried chitosan/collagen thermogels with various concentrations of RHA-Si: (**a**) 25% strain; (**b**) 50% strain; (**c**) 75% strain. Data are presented as mean ± SD. Different letters above the bars indicate statistically significant differences among groups (one-way ANOVA followed by Tukey’s HSD test, *p* < 0.05).

The compression force at a given displacement is utilized to investigate the effect of RHA-Si on the mechanical properties of the chitosan/collagen thermogel. The results are shown in Figure 7. It can be seen that the compression force increases with increasing RHA-Si concentrations from 0 to 1% *w*/*v* in the chitosan/collagen thermogel, which remains constant at 2 and 3% *w*/*v* at a displacement of 25%. However, at higher displacements (50% and 75%), the compression force of thermogels containing 2 and 3% RHA-Si decreases significantly.

The results show that the compression force of chitosan/collagen thermogels increases when silica concentration rises from 0 to 1% *w*/*v*. This means that a small amount of silica can help strengthen the thermogel. The compression force remains unchanged at 2% and 3% *w*/*v*. This indicates that increasing silica beyond 1% does not further enhance the thermogel at 25%, likely due to limitations in filler–polymer interactions and network formation within the thermogel. At higher compressions of 50% and 75% displacement, thermogels containing 2% and 3% silica exhibit a noticeable decrease in compression force. This means that high amounts of silica may reduce the thermogel’s flexibility and weaken it under large deformation [49,50].

These findings align with previous reports that RHA-Si can improve mechanical properties in specific polymer matrices such as natural rubber due to enhanced polymer–filler interactions [51,52]. However, RHA-Si may exhibit limited or adverse effects in polymers like polypropylene and EPDM, primarily due to compatibility issues arising from the hydrophilic nature of RHA-Si and the hydrophobic characteristics of these polymers unless surface treatments or compatibilizers are applied [53].

## 4. Conclusions

In this study, thermosensitive chitosan/collagen hydrogels were successfully prepared and reinforced with rice husk ash silica (RHA-Si). The chemical analysis showed that adding RHA-Si did not alter the main functional groups of the thermogels but caused slight shifts in the FTIR bands due to hydrogen bond interactions. The morphology of the freeze-dried thermogels changed with the silica content. RHA-Si helped form larger, more spherical pores at specific concentrations, although the effect was not linear. Rheological tests indicated that silica increased the storage modulus (G′), making the thermogel stiffer. However, the Payne effect weakened the network at high strain. The compression test revealed that a small amount of silica (1% *w*/*v*) improved the thermogel’s strength at low deformation. In contrast, too much silica (2–3% *w*/*v*) at high deformation made the thermogel less flexible and reduced its strength. Freeze-drying may alter pore morphology and mechanical response; rheology supported the network characteristics. These findings suggest that 1% *w*/*v* RHA-Si is the optimal concentration for balancing structural reinforcement with mechanical flexibility in chitosan/collagen thermogels. This optimized formulation may be especially suitable as an injectable scaffold for soft tissue regeneration, providing adequate stiffness to support tissue integration while maintaining sufficient flexibility to accommodate physiological deformation.

## Figures and Tables

**Table 1:** Chemical composition and percentage of RHA-silica powder determined by X-ray fluorescence (XRF).

Element	Amount (% *w*/*w*)
Si	96.91 ± 0.09
Ca	1.56 ± 0.06
P	0.321 ± 0.016
S	0.230 ± 0.011
Fe	0.220 ± 0.011
Mg	0.026 ± 0.010
K	0.183 ± 0.0091
Na	0.156 ± 0.0078
Mn	0.0709 ± 0.0035
Al	0.0443 ± 0.0062
Zn	0.0323 ± 0.0016
Ti	0.0270 ± 0.0014
Cr	0.0246± 0.0013
Cu	0.0085 ± 0.0010
Sr	0.0065 ± 0.0011

**Table 2:** Maximum mid-pore size range and standard deviation of structure separation (SD_St.Sp_) of the freeze-dried chitosan/collagen thermogels with various RHA-Si concentrations.

RHA-Si Concentration (% *w*/*v*)	Maximum Mid-Pore Size Range (mm)	SD_St.Sp_ (mm)
0	0.07	0.04
0.5	0.13	0.07
1	0.20	0.15
2	0.12	0.20
3	0.20	0.11

**Table 3:** Porosity and connectivity of the freeze-dried chitosan/collagen thermogels with various RHA-Si concentrations.

RHA-Si Concentrations (% *w*/*v*)	Porosity (%)	Connectivity Density (1/mm^3^)
0	39.41	590.64
0.5	64.81	280.52
1	74.22	144.80
2	69.47	203.03
3	70.68	175.34

## Data Availability

Data are contained within the article.
